# Investigating the relationship between neighbourhood characteristics, perceived social support and psychological wellbeing in Spanish adolescents

**DOI:** 10.1038/s41598-025-22753-1

**Published:** 2025-11-06

**Authors:** Blanca Piera Pi-Sunyer, Giacomo Bignardi, Gonzalo García-Baquero, Payam Dadvand, Martine Vrijheid, Mònica Guxens, Ana Esplugues, Juana Maria Delgado-Saborit, Jesús Ibarluzea, Sarah-Jayne Blakemore, Mikel Subiza-Pérez

**Affiliations:** 1 Department of Psychology, University of Cambridge, Cambridge, UK; 2 Social, Genetic and Developmental Psychiatry Centre, King’s College London, London, UK; 3 Faculty of Biology, University of Salamanca, Salamanca, Spain; 4 ISGlobal, Barcelona, Spain; 5 Universitat Pompeu Fabra, Barcelona, Spain; 6 Spanish Consortium for Research on Epidemiology and Public Health (CIBERESP), Madrid, Spain; 7 Department of Child and Adolescent Psychiatry/Psychology, Erasmus MC, University Medical Center, Rotterdam, the Netherlands; 8 ICREA, Barcelona, Spain; 9 Department of Nursing, Universitat de València, València, Spain; 10 Epidemiology and Environmental Health Joint Research Unit, FISABIO, Universitat Jaume I - Universitat de València, València, Spain; 11 Department of Medicine, Universitat Jaume I, Castellón de la Plana, Spain; 12 Biogipuzkoa Health Research Institute Environmental Epidemiology ES, Donostia-San Sebastián, Spain; 13 Faculty of Psychology, University of the Basque Country, Donostia-San Sebastián, Spain; 14 Institute of Cognitive Neuroscience, University College London, London, UK; 15 Group of Public Health and Environmental Epidemiology,, Biogipuzkoa Health Research Institute, Donostia-San Sebastian, Spain; 16 Department of Clinical and Health Psychology and Research Methods, University of the Basque Country UPV/EHU, Donostia-San Sebastián, Spain; 17 Bradford Institute for Health Research, Bradford, UK

**Keywords:** Human behaviour, Risk factors

## Abstract

The rapid urbanisation of many countries has raised important questions about how neighbourhood characteristics affect wellbeing, particularly during adolescence, a period of vulnerability to mental health conditions. This study investigated how population density, neighbourhood deprivation, green space availability and residential greenness are related to psychological wellbeing in adolescence, as well as the role of perceived social support and autonomy in this relationship. Using data from 970 participants aged 9–17 years in the INMA (Infancia y Medio Ambiente) cohort in Spain, collected between 2016 and 2022, we applied a set of linear mixed effects models to examine these associations, and also explored potential differences by age, sex and family affluence. The results showed an association between higher residential greenness and better psychological wellbeing in older adolescents (15–17 years). This association was partially mediated by greater family support and feelings of autonomy. In addition, there was an association between higher population density and worse psychological wellbeing in older adolescents. No significant associations were found between neighbourhood deprivation, green space availability and psychological wellbeing. Finally, the relationships reported in this study were not modified by family affluence. These findings suggest that neighbourhood residential greenness could contribute to feelings of family support and autonomy, which contribute to important aspects of psychosocial adaptation during adolescence.

**Supplementary Information:**

The online version contains supplementary material available at 10.1038/s41598-025-22753-1.

## Introduction

The last decades have been characterised by the rapid urbanisation of several parts of the world^1^. Consequently, recent research has explored the effect of growing up in urbanised environments on physical, social and psychological development^2–6^. Children and young people living in cities often have more access to health and social care services, better transportation, and better education opportunities compared to those living in rural areas^1^. However, they are also exposed to higher air and noise pollution and heat, fewer natural or green spaces, limits to personal space, and greater crime rates and socioeconomic disparities^1,7^. Adolescence is a sensitive period for the effect of the living environment on social and psychological wellbeing, and of vulnerability to the emergence of socioemotional disorders^8,9^. In this study, we sought to investigate the relationship between neighbourhood characteristics, perceived social support, and psychological wellbeing during adolescence.

Growing literature has identified compact urban forms as an important component of sustainable urbanisation^10^. Notably, however, living in densely populated environments (an element of compact urban forms) has also been related to chronic stress processing and greater symptoms of anxiety and psychotic disorders in adults^11–14^. While population density may contribute to wellbeing by offering greater opportunities for socialisation and establishing connections, this does not always translate to subjective experiences of social and psychological wellbeing^15–20^. Some studies have also reported weaker social relationships in high-density neighbourhoods^20–22^ and more feelings of insecurity and negative emotions towards neighbourhoods^17,23^. While most studies have included adult samples, living in environments that facilitate or hinder social cohesion might be particularly impactful for adolescents, as changes to their social environment influence their mood and anxiety to a greater extent than in other age groups^24,25^.

Urban neighbourhood disadvantage has also been associated with higher prevalence of emotional difficulties in young people^12,26–28^, with greater violence exposure and antisocial behaviour from peers contributing to higher depressive symptoms in adolescents living in more deprived urban neighbourhoods^29^. Other studies have found neighbourhood unpleasantness, low maintenance and perceptions of safety and crime to be associated with reduced independent mobility and exploration of neighbourhood spaces^30–32^. In turn, several indicators of neighbourhood deprivation (e.g. perceptions of safety, housing insecurity, and concentrated poverty) have been proposed to hinder social connection, such as increasing peer problems^33,34^ and feelings of loneliness^32^.

In contrast, natural features of neighbourhoods can positively contribute to young people’s wellbeing^35,36^. In fact, many guidelines now recommend having at least 3 well-established trees in view from homes, schools and places of work; no less than a 30% tree canopy in neighbourhoods; and no more than 300 m to the nearest public green space from the residential address^38–40^. Several mechanisms have been proposed to explain the beneficial health-related impact of urban green areas. For example, the availability of recreational green spaces is related to physical activity, stress reduction, and attention restoration in children^4,36,41^, and a higher percentage of tree canopy and tree diversity can have the potential to mitigate the impact of air pollution, heat and noise^3,42^. In addition, incorporating well maintained green spaces and green cover in neighbourhood design can also improve perceptions of safety and neighbourhood pleasantness^44^. Several studies have also shown that neighbourhoods with more green cover are associated with enhanced social cohesion and community building across the lifetime^16,45^. While evidence is mixed for child and adolescent samples^46,47^, one study showed that more frequent use of green spaces was associated with social connectedness with friends (quantity and time spent) in young people aged 10–18, and this relationship was particularly strong for adolescents aged 14 years and over^48^.

In line with this, neighbourhood characteristics may have different effects at different points in adolescence, although the evidence is not yet clear on which stage is most vulnerable. One reason to expect this is that exposure to urban stressors, such as noise, crowding, pollution, deprivation and safety concerns, can have cumulative effects on wellbeing. The longer young people are exposed to these stressors, the more likely they are to experience heightened psychological strain and poorer overall health^5^. Conversely, the prolonged availability of positive neighbourhood resources, such as access to green spaces, recreational facilities and supportive community environments, has been linked to improvements in health and wellbeing^50^. Because these effects accumulate over time, and adolescence is a sensitive period of development in which environmental input is particularly important, the impact of neighbourhood characteristics may become especially apparent in the psychological and social wellbeing of older adolescents^34,48^. In contrast, sociocognitive skills that underlie social stress processing and emotional regulation continue to develop throughout adolescence^51^. As a result, less developed sociocognitive skills earlier in adolescent development might heighten the susceptibility to the negative effects of urban stressors (e.g. experiences of exclusion) and the positive effects of natural areas (e.g. through physical activity and emotion regulation). Taken together, there might be age-related differences in the relationship between neighbourhood characteristics and social and psychological wellbeing, but the specific direction of these speculations requires further research.

Relatedly, some young people may encounter greater barriers in accessing health-promoting resources, depending on their family affluence or sex. For example, young people from disadvantaged backgrounds often have reduced access to healthcare services (e.g., private healthcare, specialised mental health support) and fewer opportunities to participate in structured, enriching activities such as after-school clubs, sports teams or summer programmes^52^. These opportunities are more readily available to young people from more affluent families, who can afford fees, transportation and equipment. As a result, we could speculate that the detrimental effects on wellbeing of neighbourhood factors such as a lack of green space and high neighbourhood deprivation might disproportionately affect young people who have less access to—and less ability to compensate from—specialised mental health support and enriching activities^53^. In addition, sex-related differences also shape how young people engage with their neighbourhood environment. Girls often face greater barriers to using and exploring public spaces independently, partly due to heightened safety concerns (e.g., fear of harassment, parental restrictions)^55^. This can limit their participation in certain forms of social and physical activity, such as using public recreational grounds, cycling or spending time with peers in parks or community spaces, especially after daylight hours^56^. In contrast, boys may be less restricted in their mobility and social use of neighbourhood spaces, which could lead to differences in peer network formation, physical activity levels and overall wellbeing. Taken together, these patterns suggest that the same neighbourhood characteristics can have uneven effects across groups.

### The current study

The current study applied linear mixed effects models to combined data from the Childhood and Environment cohort study^57^ (1492 total observations from 970 unique participants, comprising *n* = 743, ages 9–12 years; *n* = 528, ages 12–17 years; *n* = 221, 14–17 years), collected in three regions of Spain between 2016 and 2022. The first aim was to investigate whether several neighbourhood characteristics have an association with psychological and social wellbeing in adolescents. We hypothesised that higher residential greenness and green space availability would be related to higher psychological wellbeing and higher perceived social support. In addition, we hypothesised that neighbourhood deprivation would be related to lower psychological wellbeing and lower perceived social support. As the evidence relating population density to wellbeing is mixed^7^, we hypothesised that population density could be positively or negatively related to psychological wellbeing and social support.

As previous literature has consistently found social support to be related to psychological wellbeing in young people^58–60^, the second aim of this study was to investigate whether family and peer support play a role in the relationship between neighbourhood stressors and resources and psychological wellbeing. We hypothesised that higher perceived social support would mediate the relationship between higher residential greenness and green space availability and higher psychological wellbeing. We also hypothesised that lower perceived social support would mediate the relationship between neighbourhood deprivation and lower psychological wellbeing. In addition, we hypothesised that perceived social support would mediate the relationship between population density and psychological wellbeing. Note that mediation hypotheses do not imply longitudinal mediation, but instead compare variance in psychological wellbeing explained by differences in neighbourhood characteristics and perceived social support.

Finally, the impact of social and environmental settings on wellbeing might vary according to development and sex (i.e. age and sex differences)^35,48^, and access to health-promoting resources could mitigate the impact of living environments (i.e. family affluence differences)^61,62^. As such, our third aim was to explore, in a series of exploratory models, whether the relationship between neighbourhood characteristics, perceived social support and psychological wellbeing differ according to demographic characteristics, including age, sex and family affluence.

## Methods

### Sample

The sample included participants ages 9–17 years who are part of the INMA project (www.proyectoinma.org)^57^. The INMA project investigates the effects of environmental exposures on physical, social, and neuropsychological development from conception onwards. Between 2004 and 2008, 2644 women in the first trimester of pregnancy (over 16 years of age) were recruited from the public health service in several Spanish regions, including Gipuzkoa, Sabadell and Valencia (see Dadvand et al., 2012 for biogeographic details of regions)^63^. Follow-up data from families and children has been collected sequentially every two to three years, with slight age and time variations by region. The ethics committees of the hospitals involved in each region approved the project and supervised that the project was performed in accordance with their guidelines, and informed consent was obtained from all parents in each follow-up.

In the current study, we used combined data from the following regions: Gipuzkoa, 358 participants who took part in 2018–2019 (ages 10–12 years) and 261 participants who returned in 2021 (ages 12–14 years); Sabadell, 385 participants who took part in 2016–2018 (ages 9–12 years) and 267 participants who returned in 2020–2022 (ages 13–17 years); and Valencia, 221 participants who took part in 2019–2022 (ages 14–17 years; see Table S1 in supplemental material SM1 for sample differences between regions). Note that participants from Valencia did not have available social and psychological wellbeing questionnaire data at Time 1 and were therefore only included at Time 2. Participants who did not have complete data in all measures were excluded from the study (see missing data details in Table S2, Table S3 and Figure S1 in supplemental material SM1). There was a total of 1492 observations in the sample (from 970 unique participants).

## Measures

### Neighbourhood characteristics

The first neighbourhood characteristic was residential greenness, calculated using the Normalized Difference Vegetation Index (NDVI). The NDVI is a greenness measure derived using satellite imagery (see further details in supplemental material SM2) with values ranging from − 1 to 1: low values (0–0.1) indicate areas of barren rock, sand or snow; moderate values (0.2–0.5) indicate sparse vegetation (e.g. grasslands), and high values (0.6–1) indicate dense vegetation^64^. Negative values correspond to water and were removed before calculating residential greenness. Residential greenness was computed as the 5-year NDVI average in the greenest seasons only (May through August) in a 300 m buffer surrounding the home address. We use a 300 m buffer as this has been found to be an appropriate marker of residential surrounding greenness^65^ and is predictive of health outcomes in other studies using the INMA data^66,67^. Higher NDVI scores represent greater percentage of residential greenness.

The second neighbourhood characteristic was green space availability, computed as a binary measure measuring access to major green spaces (e.g. parks or countryside) surrounding the home address. This measure was obtained by calculating the straight-line distance from the home to the nearest green space with an area greater than 5000 m^2^ using the Europe-wide Urban Atlas^68^. Green space availability was determined by whether there was a major green space available within a 150 m buffer of the home address (1 = yes, 0 = no)^69^. We use a 150 m buffer in this study because 85.5% of the sample had a major green space available within a 300 m buffer, which is the distance recommended by the World Health Organisation^39^.

The third neighbourhood characteristic was neighbourhood deprivation, which is an index of deprivation derived from the 2011 Population and Housing Census of Spain^70^. This deprivation index is computed from six socioeconomic indicators: percentage of unemployed population, percentage of manual workers, percentage of casual workers, percentage of youth without education and number of homes without access to the internet. The index was classified into ordered tertiles (1 = low deprived, 2 = medium deprived, 3 = high deprived).

The last neighbourhood characteristic was population density, computed as the number of inhabitants per square kilometre surrounding the home address. This measure was derived as the point-to-raster assignment between geocodes and population density grid maps (250 m^2^ resolution) obtained from the 2015 Global Human Settlement Layer (GHS-POP R, 2015). Scores were transformed to 100 inhabitants per square kilometre for interpretability. Higher scores denote higher population density.

### Household-level socioeconomic measures

This study also included two household-level socioeconomic measures: family affluence and maternal education. Family affluence was collected at birth using the EU Statistics on Income and Living Conditions (EUSLIC)^72^. The EUSLIC is a measure of net disposable household income, standardised for household composition and log-transformed to better approximate a normal distribution^73^. Higher values denote higher family affluence. In addition, this study used the International Standard Classification of Education 1997 to classify maternal education during pregnancy. This is a measure of the highest level of education completed by the mother and is ordered in three categories (1 = primary or no education, 2 = secondary education, 3 = university education).

### Psychological wellbeing and perceived social support measures

The INMA study collected data on the self-reported 27-item version of the Kidscreen (Spanish version)^74^, which is a measure of health-related quality of life developed by a collaboration of 13 European countries^76–78^. The Kidscreen-27 includes five subscales measuring different dimensions of quality of life: physical wellbeing, psychological wellbeing, autonomy & parent relations, peers and social support, and school environment. All items are rated on a five-point response scale (0 = “not at all” to 4 = “extremely” or 0 = “never” to 4 = “always”). Items on each subscale are aggregated to create sum scores for each dimension which are later transformed into T-scores ranging from 0 to 100 using population norms^74^. In this study, we use the psychological wellbeing subscale, which measures positive emotions and life-satisfaction (7 items); the autonomy & parental relations subscale, which measures the quality of interactions with the family and feelings of autonomy (7 items); and the peers and social support subscale, which measures the quality of relations with peers (4 items). Higher T-scores on these subscales in general denote greater psychological wellbeing, family support & autonomy and peer support, respectively. This tool has been used and validated in several European samples in children aged 8–18 years old^75^.

### Statistical analysis

#### Main analyses

First, to determine whether perceived social support and psychological wellbeing replicated previous literature^60^, *psychological wellbeing*,* family support & autonomy* and *peer support* were regressed separately on *age* and *family affluence*, both treated as continuous measures, and *sex*, treated as a dichotomous measure (female = 0, male = 1). In addition, *psychological wellbeing* was regressed separately on *family support & autonomy* and *peer support*. Note that these analyses do not test any hypotheses but rather check that the data replicate previous literature.

Second, to investigate the effect of neighbourhood characteristics on psychological wellbeing, a set of models regressed *psychological wellbeing* on the following neighbourhood characteristics (separately): *residential greenness* and *population density*, both treated as continuous measures; *green space availability*, treated as a binary measure (1 = yes, 0 = no); and *neighbourhood deprivation*, ordered in tertiles (1 = low deprived, 2 = medium deprived, 3 = high deprived). In addition, to investigate the effect of neighbourhood characteristics on perceived social support, another set of models regressed *family support & autonomy* and *peer support* separately on each predictor.

Third, to determine whether the relationship between the neighbourhood characteristics and psychological wellbeing was explained by perceived social support in our data, we re-ran those models that showed a relationship between the neighbourhood characteristics and psychological wellbeing, adjusting for *family support & autonomy* and *peer support* (separately). Adjusting for a mediator (i.e. controlling for an indirect effect) allows us to estimate the direct effect of a predictor on an outcome measure (direct effect = total effect–indirect effect)^78^. According to this mediation literature, a mediator is likely to have a role on the relationship between the predictor and an outcome if the direct effect is weakened compared to the total effect. Given the limitations of applying mediation analyses on hierarchical mixed models with complex clusters (e.g. 3-level structures), we did not calculate inferential statistics for indirect effects.

Using the R statistical software (R version 4.3.2)^79^, the combined data (1492 observations) were modelled in a set of linear mixed effects models^80^ using the *lme4* (version 1.1–35.1)^81^ and *lmerTest* packages (version 3.1-3.1)^82^ with participant and region as random effects. Robust causal inference methodology was applied to determine the minimal covariate adjustment sets for each model by using a Directed Acyclic Graph (DAG; see Table S4, Table S5, Figure S2 and Figure S3 in supplemental material SM3 for details of the DAG and model-specific adjustments). Main effects and interactions (see below) were inspected using omnibus *F-tests* with Kenward-Roger approximations for degrees of freedom. All continuous measures were standardised (centred and scaled) prior to analysis.

### Effect modification

We additionally explored whether demographic characteristics modify the relationship between neighbourhood characteristics, perceived social support and psychological wellbeing. To do so, we re-ran the models described in the main analyses, additionally interacting the neighbourhood characteristics with one of the following predictors: *age, sex* and *family affluence*. Any significant interactions were further inspected using post-hoc marginal mean-trend estimations with Kenward-Roger approximations for degrees of freedom^83^. Specifically, we explored whether pathways described in the main analyses varied with *sex*, and at the 25th, 50th and 75th percentiles of *age* and *family affluence*, using the *emmeans* and the *emtrends* function of the *emmeans* package (version 1.10.0)^84^. Scripts for the statistical analysis can be found in the Open Science Framework (https://osf.io/d24kt/).

### Sensitivity analyses

Model diagnostics of all linear mixed effects model residuals approximated a normal distribution, but homogeneity of variance and linearity assumptions could be violated towards the extremes of the scales, potentially due to the presence of influential observations. Therefore, in a first set of sensitivity analyses, we re-ran all models excluding potential influential observations. In addition, to adjust for period-specific influences in the relationships, we re-ran a second set of sensitivity analyses additionally including *timepoint* (0,1) as a fixed effect to all models. Finally, to adjust for selective attrition of the missing data, we re-ran a third set of sensitivity analyses adjusting for participant-level weights derived from the inverse probability of inclusion predicted by age, family affluence, maternal education, timepoint and region. Omnibus tests reported in the results section are robust to sensitivity analyses adjusting for the presence of influential observations, period-specific effects and selective attrition, unless otherwise stated.

## Results

**Fig. 1:**
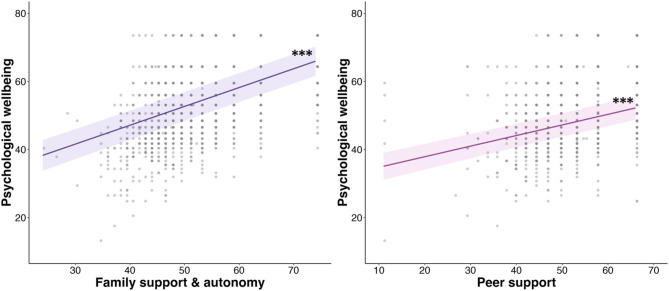
Relationship between perceived social support and psychological wellbeing. Dots show participant psychological wellbeing as a function of perceived social support, including family support & autonomy (left panel) and peer support (right panel). Lines of best fit show the main effect of family support & autonomy (in purple; left panel) and peer support (in pink; right panel) on psychological wellbeing, as estimated by the linear mixed effects models. The shaded areas are the 95% confidence intervals. The asterisks indicate significant main effects of the perceived social support measures: ***p <.001.

Descriptive statistics are reported in Table 1. The results of the linear mixed effects models investigating the relationships between demographic characteristics, psychological wellbeing and perceived social support are presented in supplemental material SM5 (see Table S6 and Figure S4). Summary statistics and post-hoc comparisons of significant main effects showed that psychological wellbeing was lower in older participants (*β*_standardised_ = − 0.49, 95%CI [− 0.54 − 0.44], *p* <.001) and in females compared to males (contrast_Female−Male_ = − 0.23, SE = 0.05, *p* <.001). In addition, psychological wellbeing was associated with higher family support & autonomy (*β*_standardised_ = 0.48, 95%CI [0.44 0.52], *p <*.001; Fig. 1, left panel) and higher peer support (*β*_standardised_ = 0.28, 95%CI [0.24 0.32], *p* <.001; Fig. 1, right panel). The relationship between peer support and psychological wellbeing was stronger in females than males (contrast_Female−Male_=0.10, 95%CI [0.02 0.17], *p* =.009) and increased with family affluence (*β*_standardised_ = 0.06, 95%CI [0.03 0.10], *p <*.001). The models also showed significant associations between age and family support & autonomy, age and peer support, and family support & autonomy and sex. However, these were not robust to adjusting for period-specific effects (see Table S7 in supplemental material SM6 for full details and results of sensitivity analyses).

**Table 1:** Descriptive statistics of the sample.

Measures	Total Sample *N* = 1492 9–17 years	Time 1 *n* = 743 9–12 years	Time 2 *n* = 749 12–17 years
Age			
Range	9.9–17.5	9.9–12.4	12.7–17.5
Mean (SD)	12.9 (2.1)	10.9 (0.5)	14.9 (1.0)
Sex			
Female	781 (52.3%)	382 (51.4%)	399 (53.3%)
Male	711 (47.7%)	361 (48.6%)	350 (46.7%)
Family affluence			
Range	6.2–7.8	6.2–7.8	6.2–7.8
Mean (SD)	7.2 (0.3)	7.2 (0.3)	7.2 (0.3)
Maternal education			
Primary or no education	270 (18.1%)	135 (18.2%)	135 (18.0%)
Secondary education	592 (39.7%)	300 (40.4%)	292 (39.0%)
University education	630 (42.2%)	308 (41.4%)	322 (43.0%)
Psychological wellbeing			
Range	13.2–73.5	33.2–73.5	13.2–73.5
Mean (SD)	52.4 (10.0)	56.5 (8.9)	48.4 (9.4)
Family support & autonomy			
Range	24.2–74.4	26.6–74.4	24.2–74.4
Mean (SD)	52.5 (8.7)	53.2 (8.7)	52.0 (8.6)
Peer support			
Range	11.2–66.3	11.2–66.3	11.2–66.3
Mean (SD)	54.4 (8.9)	55.8 (8.5)	53.0 (9.0)
Population density			
Range	0–280.3	0–258.2	0–280.3
Mean (SD)	74.0 (43.6)	78.2 (41.9)	69.8 (44.8)
Residential greenness			
Range	12.2–84.5	12.2–84.5	12.2–82.8
Mean (SD)	34.1 (15.6)	35.6 (15.7)	32.6 (15.3)
Neighbourhood deprivation			
Low deprived	869 (58.2%)	454 (61.1%)	415 (55.4%)
Medium deprived	540 (36.2%)	251 (33.8%)	289 (38.6%)
High deprived	83 (5.6%)	38 (5.1%)	45 (6.0%)
Green space availability			
Yes	910 (61.0%)	450 (60.6%)	460 (61.4%)
No	582 (39.0%)	293 (39.4%)	289 (38.6%)

The table shows descriptive statistics of the sample, including age (in years) and family affluence (log-transformed household disposable income), sex (female, male), maternal education (primary or no education, secondary education, university education), psychological wellbeing, family support & autonomy and peer support (all T-scores; 0–100) and neighbourhood characteristics, including population density (in 100 inhabitants per square kilometre), residential greenness (in percent cover in a 300 m buffer), neighbourhood deprivation (low deprived, medium deprived, high deprived) and green space availability (yes, no). The columns show the range, mean and standard deviations (SD) for each continuous measure, and frequencies for binary and ordinal measures, for the total sample (left column), for the sample at Time 1 (middle column) and for the sample at Time 2 (right column).

### Relationship between neighbourhood characteristics and psychological wellbeing

The results of the linear mixed effects models investigating the relationship between the neighbourhood characteristics and psychological wellbeing, as well as exploratory interactions with demographic characteristics, are presented in Table 2. Significant interactions are presented in bold.

**Table 2:** Results of the linear mixed effects models for psychological wellbeing.

Predictor measure	Effect modification	F statistics (DF)	*p*-value
Residential greenness		*F*(1,247.16) = 0.03	0.866
	**Age**	***F*(1**,**829.03) = 8.95**	**0.003**
	Sex	*F*(1,853.14) = 1.54	0.215
	Family affluence	*F*(1,864.52) = 0.44	0.506
Population density		*F*(1,923.66) = 0.11	0.740
	**Age**	***F*(1**,**759.01) = 9.59**	**0.002**
	Sex	*F*(1,939.60) = 1.69	0.194
	Family affluence	*F*(1,992.83) = 0.52	0.471
Neighbourhood deprivation		*F*(2,1039.61) = 0.62	0.540
	Age	*F*(2,1121.71) = 1.26	0.283
	Sex	*F*(2,1044.92) = 0.67	0.511
	Family affluence	*F*(2,1071.53) = 2.33	0.098
Green space availability		*F*(1,935.77) = 0	0.966
	Age	*F*(1,1035.62) = 0.19	0.667
	Sex	*F*(1,894.24) = 0.30	0.583
	Family affluence	*F*(1,956.05) = 0.04	0.839

Summary of the linear mixed effects models investigating the relationship between neighbourhood characteristics (first column) and psychological wellbeing, including any interactions with effect modifying measures (second column). Significant *F*-tests (fourth column) and associated p-values (last column) are presented in bold.

### Residential greenness

**Fig. 2:**
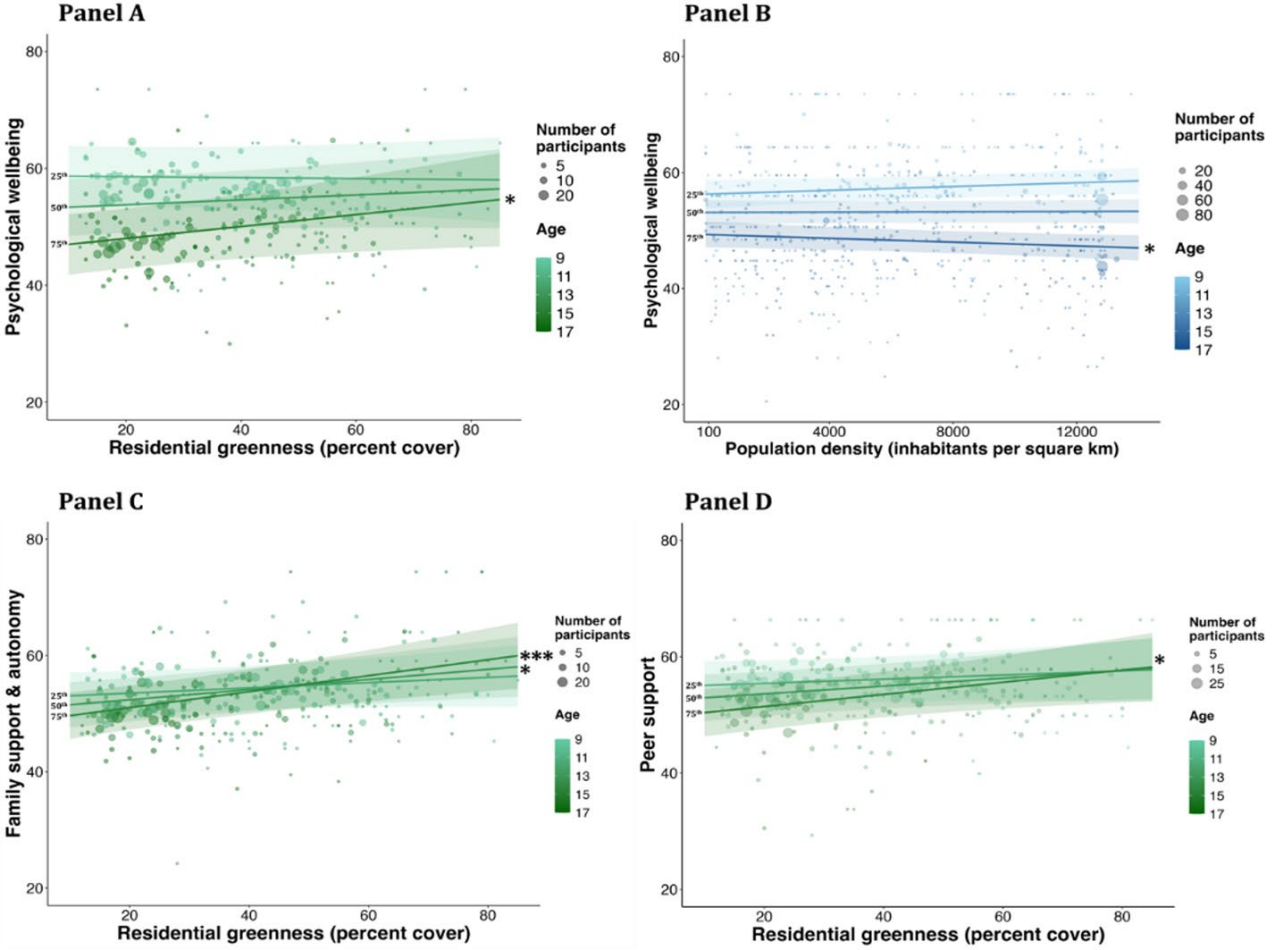
Age-related differences in the relationship between neighbourhood characteristics, psychological wellbeing and perceived social support *Top panels.* Circles show grand means of psychological wellbeing as a function of residential greenness (percent cover; Panel A) and population density (inhabitants per square km; Panel B), colour-coded by age (in years; 9–17 years; light to dark tones). *Bottom panels.* Circles show grand means of family support & autonomy (Panel C) and peer support (Panel D) as a function of residential greenness (percent cover), colour-coded by age (in years; 9–17 years; light to dark tones). *All panels.* Circle area is proportional to the number of participants; the keys show examples for reference. Lines of best fit show the marginal mean-trends at the 25th (10.84 years; light tone), 50th (12.79 years; medium tone) and 75th (15.13 years; dark tone) percentiles of age, as estimated by the linear mixed effects models. The shaded areas are the 95% confidence interval. The asterisk indicates a significant marginal mean-trends of age: ***p <.001; *p <.05.

As reported in Table 2, the analyses of the linear mixed effects models showed no main effect of residential greenness on psychological wellbeing. However, a separate exploratory analysis showed a positive interaction between residential greenness and age (*β*_stadardised_=0.08, 95%CI [0.03 0.13], *p* =.003). In support of our hypotheses, exploratory post-hoc marginal mean-trend estimations at the 75th percentile of age (15.13 years) showed that the relationship between higher residential greenness and higher wellbeing in older individuals was small and significant (Q75: *β*_standardised_=0.15, 95%CI [0.01 0.30], *p =*.038; Fig. 2, Panel A). In contrast, exploratory post-hoc marginal mean-trend estimations at the 25th (10.84 years) and 50th percentile of age (12.79 years) did not show a relationship between residential greenness and psychological wellbeing in younger individuals (Q25: *β*_standardised_= − 0.01, 95%CI [− 0.14 0.12], *p* =.896; Q50: *β*_standardised_=0.06, 95%CI [− 0.06 0.19], *p* =.312). Finally, separate exploratory analyses did not show an interaction between residential greenness and sex, or residential greenness and family affluence, on psychological wellbeing. These models included population density, neighbourhood deprivation, green space availability, maternal education and family affluence as covariates, as determined by the DAG minimum covariate adjustment set.

### Population density

As reported on Table 2, the analyses of the linear mixed models showed no main effect of population density on psychological wellbeing. However, a separate exploratory analysis showed a small negative interaction between population density and age (*β*_standardised_ = − 0.07, 95%CI [− 0.12 − 0.03], *p* =.002). Exploratory post-hoc marginal mean-trend estimations at the 25th (10.84 years), 50th (12.79 years) and 75th percentiles of age (15.13 years) showed that, in line with our hypothesis, there was a weak and significant relationship between higher population density and lower wellbeing in older individuals (*β*_standardised_ = − 0.07, 95%CI [− 0.14 0], *p* =.044; Fig. 2, Panel B). Population density was not related to psychological wellbeing in younger individuals (Q25: *β*_standardised_ = 0.07, 95%CI [− 0.01 0.15], *p* =.090; Q50: *β*_standardised_ = 0.01, 95%CI [− 0.06 0.07], *p* =.845). Note however that the association between higher population density and psychological wellbeing in older adolescents was weak and no longer significant in sensitivity analyses adjusting for period-specific effects (*β*_standardised_ = − 0.07, 95%CI [− 0.14 0], *p* =.053; see SM6 and Table S7 for full details and results of sensitivity analyses). Further, separate exploratory analyses did not show an interaction between population density and sex, or population density and family affluence, on psychological wellbeing. These models included maternal education and family affluence as covariates, as determined by the DAG minimum covariate adjustment set.

### Neighbourhood deprivation and green space availability

As reported in Table 2, the analyses of the linear mixed effects models showed that neighbourhood deprivation and green space availability were not related to wellbeing. In addition, separate exploratory models showed no differences in these relationships according to age, sex or family affluence. Therefore, our hypotheses were not supported for neighbourhood deprivation and green space availability.

### Relationship between neighbourhood characteristics and perceived social support

The results of the linear mixed effects models investigating the relationship between the neighbourhood characteristics and perceived social support, as well as exploratory interactions with sociodemographic characteristics, are presented in Table 3. Significant main effects and interactions are presented in bold.

**Table 3:** Results of the linear mixed effects models for perceived social support.

Predictor measure	Outcome measure	Effect modification	F statistic (DF)	*p*-value
Residential greenness	**Family support & autonomy**		***F*(1**,**7.96) = 5.73**	**0.044**
		**Age**	***F*(1**,**760.70) = 7.66**	**0.006**
	Peer support		F(1,6.02) = 3.14	0.127
		Age	F(1,780.55) = 3.80	0.052^†^
Population density	Family support & autonomy		*F*(1,802.83) = 0.05	0.816
		Age	*F*(1,913.02) = 1.36	0.244
	Peer support		*F*(1,353.34) = 0.48	0.491
		Age	*F*(1,133.36) = 1.44	0.233
Neighbourhood deprivation	Family support & autonomy		*F*(2,1068.44) = 0.21	0.811
	Peer support		*F*(2,1048.63) = 0.84	0.430
Green space availability	Family support & autonomy		*F*(1,973.55) = 0.32	0.572
	Peer support		*F*(1,955.11) = 0.11	0.746

Summary of linear mixed effects models investigating the relationship between neighbourhood characteristics (first column) and perceived social support (second column), including any interactions with effect modifying measures (third column). Significant *F*-tests (fourth column) and associated p-values (last column) are presented in bold. The symbol (†) indicates a weak and nonsignificant association.

### Residential greenness

#### Family support and autonomy

As reported in Table 3, the analyses of the linear mixed effects model showed a significant main effect of residential greenness on family support & autonomy. In line with our hypotheses, higher residential greenness was in general related to higher family support & autonomy (*β*_standardised_ = 0.16, 95%CI [0.06 0.26], *p* =.044). In addition, a separate exploratory analysis showed a positive interaction between residential greenness and age on family support & autonomy (*β*_standardised_ = 0.08, 95%CI [0.03 0.14], *p* =.006). In support of our hypotheses, exploratory post-hoc marginal mean-trend estimations at the 25th (10.84 years), 50th (12.79 years) and 75th percentile of age (15.13 years) showed that the overall relationship between higher residential greenness and higher family support & autonomy was small and significant for middle and older individuals (Q50: *β*_standardised_ = 0.16, 95%CI [0.01 0.30], *p* =.035; Q75: *β*_standardised_ = 0.25, 95%CI [0.08 0.41], *p* =.004), but was not significant for the youngest individuals (Q25: *β*_standardised_ = 0.08, 95%CI [− 0.07 0.23], *p* =.271; Fig. 2, Panel C). These models included population density, neighbourhood deprivation, green space availability, maternal education and family affluence as covariates, as determined by the DAG minimum covariate adjustment set.

#### Peer support

The analyses of the linear mixed effects model showed no main effect of residential greenness on peer support. A separate exploratory analysis showed a weak and nonsignificant positive interaction between residential greenness and age on peer support (*β*_standardised_ = 0.06, 95%CI [0 0.12], *p* =.052). Exploratory post-hoc marginal mean-trend estimations at the 25th (10.84 years), 50th (12.79 years) and 75th percentile of age (15.13 years) showed that, while the relationship between residential greenness and peer support was not significant in younger individuals (Q25: *β*_standardised_ = 0.06, 95%CI [− 0.08 0.21], *p* =.392; Q50: *β*_standardised_ = 0.12, 95%CI [− 0.03 0.26], *p* =.103), higher residential greenness was related to higher peer support in older individuals (Q75: *β*_standardised_ = 0.18, 95%CI [0.02 0.34], *p* =.028; Fig. 2, Panel D). The interaction between residential greenness and age was significant after adjusting for period-specific effects (*β*_standardised_ = 0.08, 95%CI [0.01 0.14], *p* =.034) and for selective attrition (*β*_standardised_ = 0.06, 95%CI [0 0.12], *p* =.047). These models included population density, neighbourhood deprivation, green space availability, maternal education and family affluence as covariates, as determined by the DAG minimum covariate adjustment set.

### Residential greenness and psychological wellbeing, adjusting for perceived social support

Follow-up analyses explored the remaining direct interaction of residential greenness and age on psychological wellbeing, after adjusting for family support & autonomy. In line with our hypotheses, the interaction between residential greenness and age on psychological wellbeing was no longer significant when the model was adjusted for family support & autonomy (*F*(1,872.84) = 2.60, *p* =.107). Therefore, higher family support & autonomy could play a role in the relationship between higher residential greenness and higher psychological wellbeing in older individuals. In addition, follow-up analyses explored the remaining interaction of residential greenness and age on psychological wellbeing, after adjusting for peer support. The interaction between residential greenness and age weakened but remained significant when the model was adjusted for peer support (*F*(1,828.62) = 4.89, *p* =.027). Therefore, higher peer support might only play a weak role in age-related differences in the relationship between residential greenness and higher psychological wellbeing in our data.

#### Population density, neighbourhood deprivation, and green space availability

Finally, contrary to our hypotheses, the linear mixed effects models investigating the relationship between population density, neighbourhood deprivation and green space availability and perceived social support did not show a relationship with family support & autonomy or with peer support. In addition, while the relationship between population density and psychological wellbeing varied according to age, the relationship between population density and perceived social support did not vary according to age.

## Discussion

The current study investigated the relationship between neighbourhood stressors and resources, perceived social support and psychological wellbeing in adolescents aged 9–17 years from the Spanish INMA cohort. In support of our hypotheses regarding residential greenness, the age-related results suggest that higher residential greenness was related to higher psychological wellbeing in older adolescents (aged 15–17 years), but not in younger children and adolescents (aged 9–14 years). In addition, higher family support and feelings of autonomy in older adolescents living in neighbourhoods with higher residential greenness played a role in this relationship. There was weak evidence that the relationship between higher residential greenness and higher peer support played a role in psychological wellbeing. In addition, the results showed age-related differences in the relationship between population density and psychological wellbeing, with older adolescents living in more densely populated neighbourhoods reporting lower wellbeing. Contrary to our hypotheses regarding neighbourhood deprivation and green space availability, there was no relationship between these neighbourhood characteristics, perceived social support, and psychological wellbeing. Finally, and in contrast to our exploratory hypotheses, none of the significant relationships varied according to sex and family affluence.

### Residential greenness, perceived social support and psychological wellbeing

Based on the growing literature indicating an association between urban living and worse physical and mental wellbeing across the lifespan^5,7,12,41^, this study investigated whether living in areas with more greenness was related to improved psychological and social wellbeing in young people. In line with our hypotheses, higher residential greenness was related to higher psychological wellbeing in older adolescents (aged 15–17 years). Given that socioemotional difficulties typically worsen throughout adolescence^8^, our findings could contribute to an understanding of how greener living environments can reduce age-related vulnerabilities in wellbeing. Conversely, and in line with literature showing mixed or no associations between green environments and cognitive and emotional development in children^85^, the relationship between residential greenness and psychological wellbeing was not significant in children and young adolescents. Previous literature has also reported age-related effects in the relationship between residential greenness and psychological wellbeing^35,48^. While the effects reported here are small, small effects are still meaningful in understanding health inequalities, as they accumulate over populations and over time^86^. It is possible that the age-related differences discussed below might become increasing relevant with rapidly changing urban environments.

There are at least a couple of developmental explanations for the results reported in this study. First, feelings of autonomy and learning how to navigate complex social environments outside the home environment are key developmental tasks for adolescents in industrialised societies^87,88^, and these are related to positive affect and social connectivity^89^. In line with our hypotheses, our findings showed that higher residential greenness was in general related to higher family support & autonomy and this relationship played a role in higher psychological wellbeing in older adolescents living in environments with higher residential greenness. Future research could extend the current findings to understand how neighbourhood design and the spatial distribution of residential greenness can contribute to feelings of autonomy in young people^44^, perhaps by enabling independent mobility and exploration, and therefore contributing to overall psychosocial adaptation of young people^2^.

Second, belonging to peer groups and social evaluative concerns are particularly salient during adolescence^25^, with quality of friendships being an important determinant of positive self-perceptions and psychosocial adaptation during this period^90,91^. In line with research suggesting that residential greenness offers more spaces to build social ties^16,46,48^, our results show a weak but nonsignificant relationship between higher residential greenness and higher peer support in older adolescents. While further evidence is needed to support a positive role of residential greenness on peer support, it is possible that socialising and connectedness benefits of neighbourhoods characterised by residential greenness could be particularly impactful later in adolescence when peer networks become increasingly important^92^.

The health-promoting association of residential greenness, psychological wellbeing and perceived social support did not extend to green space availability, measured as whether or not there was a green area larger than 5000 m^2^ within a 150 m distance from the home. While large green spaces contribute to recreational uses of urban green, the benefits to subjective social and psychological wellbeing might depend rather on the daily use of these areas, which was not captured in this measure.

### Population density and psychological wellbeing

Another aim of this study was to investigate whether population density was related to psychological wellbeing in children and young people. The results showed some support for our hypotheses, such that older adolescents (15–17 years) living in densely populated neighbourhoods showed lower psychological wellbeing than those in less densely populated neighbourhoods. This effect was weakened when adjusting for period-specific effects (*p* =.053). It is possible that the measure of population density used in this study captures a wider array of factors related to urban living that could independently contribute positively and negatively to wellbeing, weakening the overall effect. For example, densely populated urban environments are typically characterised by more traffic, noise and air pollution^1^, as well as by some positive factors, including more opportunities for participating in leisure activities^41^. Future research could investigate how specific factors in densely populated urban neighbourhoods might positively or negatively contribute to wellbeing and how sensitivities might differ throughout childhood and adolescence.

### Neighbourhood deprivation and social inequalities in perceived social support and psychological wellbeing

Contrary to our hypotheses, there was no relationship between neighbourhood deprivation, perceived social support and psychological wellbeing. In addition, family affluence was not related to differences in psychological wellbeing and young people from disadvantaged backgrounds did not benefit to a greater extent from social and health-related benefits of residential greenness and green space. These results are inconsistent with the literature on social inequalities^94,95^ and the notion that young people from lower socioeconomic backgrounds are more likely to live in neighbourhoods that accumulate risky urban exposures (e.g. less natural space^97^).

The lack of a relationship between family affluence and wellbeing might result from the fact that the main socioeconomic measures available in the INMA dataset were collected during pregnancy or in early childhood, including the measures included in this study (family affluence, maternal education and neighbourhood deprivation). Past studies using the INMA dataset have shown that parental social class, parental education, and employment-related measures collected at birth were all predictive of cognitive outcomes in children aged 5–6 years^98^ and 7–8 years^99^. In addition, maternal education at birth, but not neighbourhood deprivation, was related to differences in parent-reported internalising and externalising difficulties in children aged 6–12 years^67^. One explanation for the null results in this study is that objective measures of family affluence might not completely represent subjective experiences of socioeconomic contexts, which might be particularly important for subjective wellbeing during adolescence^100^.

### Limitations

The findings in this study should be interpreted considering certain limitations. First, some measures included in this study might have been affected by participant biases or measurement errors. For example, we used self-report measures of perceived social support and psychological wellbeing. These could have been affected by participant-specific affective states such as mood, which might have led to biased responses. Nonetheless, while the correlations between subscales were moderate to high (*r* =.37 to *r* =.57), this range is not high enough to be problematic^101^. In addition, the measures of perceived social support and psychological wellbeing were collected concurrently. Therefore, while this study investigated the effect of perceived social support on psychological wellbeing, higher psychological wellbeing could in turn influence perceived peer support, family support and feelings of autonomy, which is in line with research reversely showing better psychosocial adaptation (e.g. sociocognitive abilities) in young people with better mental wellbeing^59^. Also, our socioeconomic variables were collected at pregnancy, birth or estimated in early childhood and might only be representative of a short timeframe.

Second, while we used a directed acyclical graphic to map out the complex empirical relationships between the relevant covariates and confounder variables, it is possible that the overall small relationships found in our study reflect a combination of complex relationships from unmeasured variables and confounders not included in the causal model. For example, ethnicity and migrant status have been related to a heightened risk of emotional disorders and psychosis around Europe^102^, but there was insufficient variance to include these factors in our model. Parental mental health and genetic-related factors are also important predictors of child mental health^8,103^, and these have been shown to influence the likelihood of families migrating to urban centres^104^. In addition, while we cluster participants by region of study, a better representation of the residential neighbourhood environment would be postcodes or censual districts. Notably, however, the participants in this study might not only be exposed to residential environments, but might also be exposed to other environments (e.g. school). This is known in epidemiological research as the uncertain geographic context problem^105,106^. Future research could account for smaller and diverse geographic contexts.

Finally, most of the Time 2 follow-up data was collected during or after the coronavirus pandemic, and therefore the age-related differences in the results presented in this study could have been amplified by lockdown restrictions, which were particularly strict in urban settings. To adjust for period-specific influences in our results, we ran a set of sensitivity analyses including timepoint as a fixed effect. These sensitivity analyses suggested that: (i) the age-related effect of higher residential greenness on higher psychological wellbeing and perceived social support was robust to adjustment for period-specific effects; (ii) the age-related effects were mostly in older adolescents (aged 15 years and older) and not in middle adolescents (under 15 years), who were also tested during the pandemic; and (iii) these age-related differences were not driven by greater variance in psychological wellbeing responses in older adolescents (i.e. homogeneity of variance assumptions were met). In addition, the unexpected main effect of age on lower family support & autonomy and lower peer support might have been driven by period-specific effects. Importantly, however, timepoint and age are highly correlated in our data, resulting in a high variance inflation factor. Therefore, while it remains important to understand how age-related differences can emerge in the relationship in neighbourhood characteristics and psychological and social wellbeing, our results should be interpreted in the context of the pandemic.

## Conclusion

The current study suggested that certain neighbourhood characteristics, including residential greenness and population density, may be related to adolescent wellbeing. Particularly, the findings suggested that older adolescents living in neighbourhoods with higher residential greenness experienced higher feelings of autonomy and perceived social support, and these were related to higher psychological wellbeing. In addition, we found that older adolescents living in densely populated urban environments reported lower psychological wellbeing. Future research could build on the current findings to investigate whether certain neighbourhood environments (e.g., greener neighbourhoods) might enable exploration and independence, contributing to healthy integration into friendship and larger peer groups. Finally, adopting a developmental framework, and taking into account biological, cognitive and social differences across childhood and adolescence, could be beneficial for studying population health effects in epidemiological studies, as these could disentangle how different urban exposures are related to wellbeing at different points in development (e.g. sensitive periods for specific urban stressors).

## Supplementary Information

Below is the link to the electronic supplementary material.

Supplementary Material 1

## Data Availability

Analyses scripts and noted results for the analyses presented in this study are available on the Open Science Framework (https://osf.io/d24kt/). In order to protect participant confidentiality and due to legal and ethical regulations, supporting data cannot be made publicly available. Bona fide researchers can apply for access to the INMA project director upon reasonable request via the project website (https://www.proyectoinma.org/en/contact-us/). Proposals for submissions should be sent to inma@proyectoinma.org.
