# Supplementary material for: Investigating the relationship between neighbourhood characteristics, perceived social support and psychological wellbeing in Spanish adolescents
Source: Sci Rep. 2025 Nov 6;15:38845. doi: 10.1038/s41598-025-22753-1 (PMC12592714; doi:10.1038/s41598-025-22753-1)
Supplement: Supplementary file 1 — Supplementary Material 1 [file 41598_2025_22753_MOESM1_ESM.docx]

**Supplemental Material**

**SM1. Sample and missing data**

The participants from each region of the INMA project took part at differing ages and time intervals. In addition, the nationwide collaboration meant that the measures included in the project were adapted to the interest of the research groups leading the project in each region (see more details in [www.proyectoinma.org](http://www.proyectoinma.org)). This resulted in some missing data for our analyses, including from participants who did not return at Time 2 (attrition), participants with available data at Time 2 only, and data missing at random for certain measures. To adapt the data for robust causal inference methodology, this study uses complete cases. Note that participants with missing data at Time 1 or Time 2 were only excluded from the relevant timepoint. Figure S1 summarises the number of participants with missing data for each measure and region. Table S1 summarises sample differences of participants who were included in each region. Table S2 summarises sample differences between excluded and included participants due to data missing at random. Table S3 summaries sample differences between participants who returned at Time 2 and those who did not return (attrition).


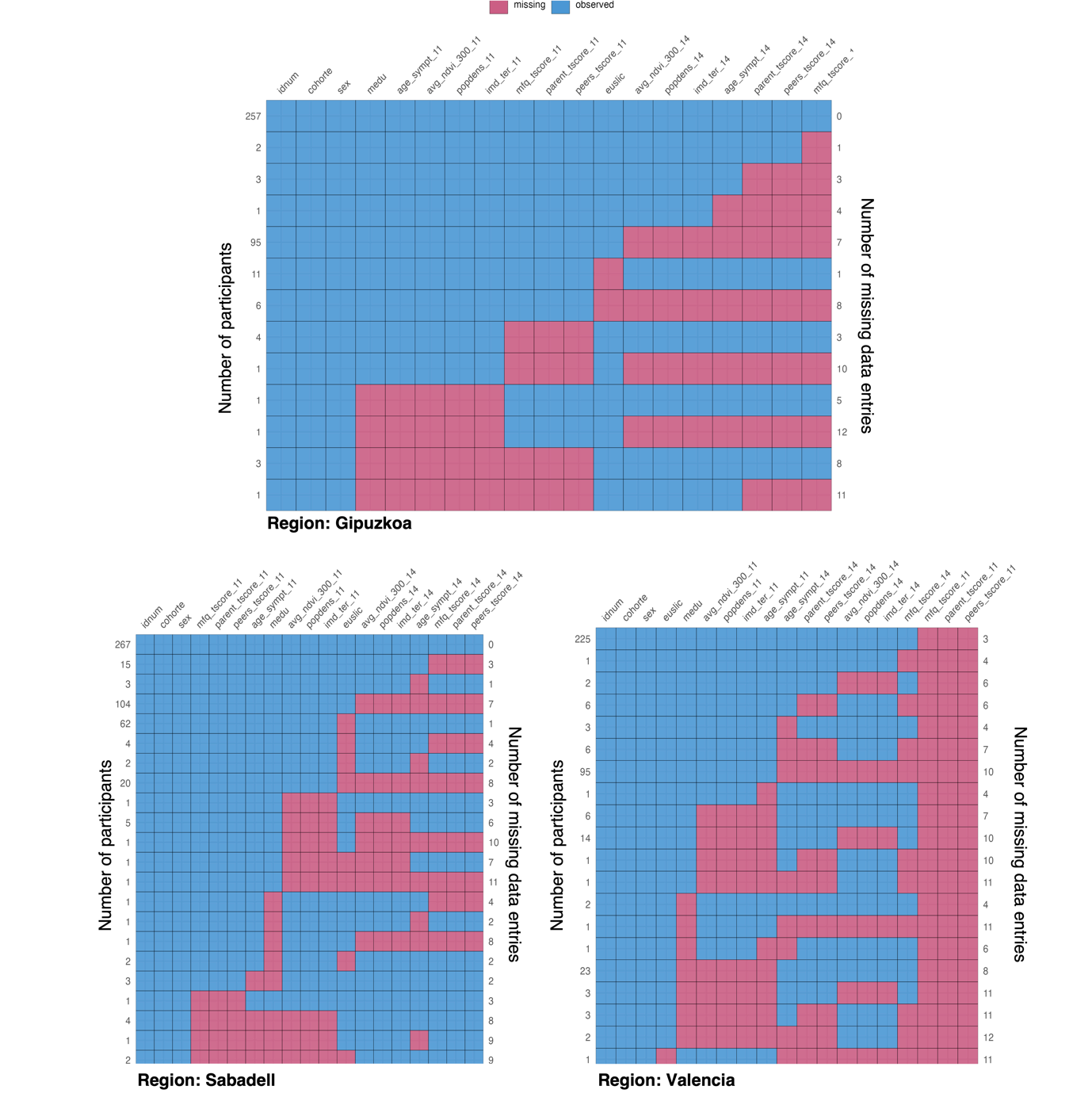


**Figure S1. Maps of missing data for each measure and region.** The plots include the observed (blue) and missing (pink) data for participants in each of the three regions, Gipuzkoa (top), Sabadell (bottom, left) and Valencia (bottom, right). The top x-axis shows the name of the measures, the left y-axis shows the number of participants with the combination of observed or missing data, and the right y-axis shows the number of missing data entries in each combination. Measures collected at Time 1 are appended with *_11* and measures collected at Time 2 are appended with *_14*. Measures without an appendix are time invariant.

idnum = participant id, cohorte = region id, medu = maternal education, age_sympt = age of the participant, avg_ndvi_300 = residential greenness, popdens = population density, imd_ter = neighbourhood deprivation, mfq_tscore = psychological wellbeing, parent_tscore = family support & autonomy, peers_tscore = peer support, euslic = family affluence.

**Table S1**

**Sample demographics of included participants**

|  | **Gipuzkoa** | **Sabadell** | **Valencia** | **Sample differences** |
| --- | --- | --- | --- | --- |
| **Time 1**  **(n=743; 9–12 years)** | **n=358** | **n=385** | **NA** |  |
| *Age*  Mean (SD) [Range]  Sample differences | 10.7 (0.3) [10.2–11.7] | 11.2 (0.5) [9.9–12.4] |  |  |
| Gipuzkoa/Sabadell |  |  |  | **t(569.81)=−16.06; *p*<.001** |
| *Family affluence*  Mean (SD) [Range]  Sample differences | 7.2 (0.3) [6.5–7.8] | 7.1 (0.3) [6.2–7.8] |  |  |
|  |  |  |  |  |
| Gipuzkoa to Sabadell |  |  |  | **t(733.69)=5.91, *p*<.001** |
| *Sex*  Female  Male  Sample differences  Gipuzkoa/Sabadell | 195 (54.5%)  163 (45.5%) | 187 (48.6%)  198 (51.4%) |  | *χ*^2^(1)=2.35; *p*=.125 |
|  |  |  |  |  |
|  |  |  |  |  |
|  |  |  |  |  |
| *Maternal education*  Primary or no education  Secondary education  University education  Sample differences | 33 (9.2%)  139 (38.8%)  186 (52%) | 102 (26.5%)  161 (41.8%)  122 (31.7%) |  |  |
| Gipuzkoa/Sabadell |  |  |  | ***χ*^2^(2)=49.26; *p*<.001** |
| **Time 2**  **(n=749; 12–17 years)** | **n=261** | **n=267** | **n=221** |  |
| *Age*  Mean (SD) [Range]  Sample differences | 13.6 (0.4) [12.7–14.4] | 15.5 (0.7) [13.2–17.5] | 15.5 (0.4) [14.7–17.0] |  |
| Gipuzkoa/Sabadell  Gipuzkoa/Valencia  Sabadell/Valencia |  |  |  | **t(472.85)=−39.13; *p*<.001**  **t(479.88)=−49.85; *p*<.001**  t(436.25)=0.71, *p*=.477 |
| *Family affluence*  Mean (SD) [Range]  Sample differences  Gipuzkoa/Sabadell  Gipuzkoa/Valencia  Sabadell/Valencia | 7.3 (0.3) [6.6–7.8] | 7.2 (0.3) [6.2 – 7.8] | 7.1 (0.3) [6.4–7.8] |  |
|  |  |  |  |  |
|  |  |  |  | **t(512.79)=4.26; *p*<.001** |
|  |  |  |  | **t(412.27)=5.91; *p*<.001** |
|  |  |  |  | t(456.74)=1.92, *p*=.055 |
| *Sex*  Female  Male  Sample differences | 149 (57.1%)  112 (42.9%) | 134 (50.2%)  133 (49.8%) | 116 (52.5%)  105 (47.5%) |  |
| Gipuzkoa/Sabadell |  |  |  | *χ*^2^(1)=0.04; *p*=.844 |
| Gipuzkoa/Valencia |  |  |  | *χ*^2^(1)=1.40; *p*=.237 |
| Sabadell/Valencia |  |  |  | *χ*^2^(1)=2.12; *p*=.146 |
| *Maternal education*  Primary or no education  Secondary education  University education  Sample differences | 23 (8.8%)  94 (36%)  144 (55.2%) | 62 (23.2%)  110 (41.2%)  95 (35.6%) | 50 (22.6%)  88 (39.8%)  83 (37.6%) |  |
| Gipuzkoa/Sabadell |  |  |  | *χ*^2^(2)=5.89; *p*=.053 |
| Gipuzkoa/Valencia |  |  |  | ***χ*^2^(2)=12.04; *p*=.002** |
| Sabadell/Valencia |  |  |  | ***χ*^2^(2)=33.59; *p*<.001** |

*Note.* The table shows age (in years) and family affluence (range, mean and standard deviation; SD), sex (female, male) and maternal education (primary or no education, secondary education, university education) for participants at Time 1 and Time 2 in each region. NA=not available. The last column presents t-test and *χ*^2^ test statistics for sample differences between regions. Significant differences are presented in bold. In general, the sample from Gipuzkoa was younger, had greater family affluence and higher maternal education than the sample from Sabadell and Valencia.

**Table S2**

**Demographics of participants who were included and excluded for missing data at random**

|  | **Included** | **Excluded** | **Difference** |
| --- | --- | --- | --- |
| **Time 1 (N = 768; 9–12 years)** |  |  |  |
| **Gipuzkoa** | **n = 358** | **n = 24** |  |
| *Age* |  |  |  |
| Mean (SD) [Range] | 10.7 (0.3) [10.2–11.7] | 10.6 (0.2) [10.2–11.1] | t(12.88)=−0.70, *p*=.486 |
| *Family affluence* |  |  |  |
| Mean (SD) [Range] | 7.2 (0.3) [6.5–7.9] | 7.3 (0.2) [7.0–7.7] | t(6.29)=1.21, *p*=.270 |
| *Sex* |  |  |  |
| Female N (%) | 195 (54.5%) | 11 (45.8%) | *χ*^2^(1)=0.37; *p*=.541 |
| Male N (%) | 163 (45.5%) | 13 (54.2%) |  |
| *Maternal Education* |  |  |  |
| Primary or no education N (%) | 33 (9.2%) | 4 (18.2%) | *χ*^2^(2)=2.46; *p*=.292 |
| Secondary education N (%) | 139 (38.8%) | 6 (27.3%) |  |
| University education N (%) | 186 (52.0%) | 12 (54.5%) |  |
| **Sabadell** | **n = 385** | **n = 110** |  |
| *Age* |  |  |  |
| Mean (SD) [Range] | 11.2 (0.5) [9.9–12.4] | 10.5 (0.6) [9.5–12.7] | **t(147.73)=−9.36, *p*<.001** |
| *Family affluence* |  |  |  |
| Mean (SD) [Range] | 7.1 (0.3) [6.2–7.8] | 7.2 (0.3) [6.7–7.7] | t(18.47)=0.91, *p*=.374 |
| *Sex* |  |  |  |
| Female | 187 (48.6%) | 52 (47.3%) | *χ*^2^(1)=0.02; *p*=.895 |
| Male | 198 (51.4%) | 58 (52.7%) |  |
| *Maternal Education N (%)* |  |  |  |
| Primary or no education | 102 (26.5%) | 26 (25.5%) | ***χ*^2^(2)=7.40; *p*=.025** |
| Secondary education | 161 (41.8%) | 30 (29.4%) |  |
| University education | 122 (31.7%) | 46 (45.1%) |  |
| **Time 2 (N = 874; 12–17 years)** |  |  |  |
| **Gipuzkoa** | **n = 261** | **n = 22** |  |
| *Age* |  |  |  |
| Mean (SD) [Range] | 13.6 (0.4) [13.2–17.5] | 13.7 (0.5) [12.9–14.4] | t(22.95)=0.23, *p*=.820 |
| *Family affluence* |  |  |  |
| Mean (SD) [Range] | 7.3 (0.25) [6.6–7.8] | 7.2 (0.2) [6.9–7.6] | t(11.35)=−0.98, *p*=.346 |
| *Sex N (%)* |  |  |  |
| Female | 149 (57.1%) | 9 (40.9%) | *χ*^2^(1)=1.55; *p*=.214 |
| Male | 112 (42.9%) | 13 (59.1%) |  |
| *Maternal Education N (%)* |  |  |  |
| Primary or no education | 23 (8.8%) | 2 (11.8%) | *χ*^2^(2)=1.26; *p*=.532 |
| Secondary education | 94 (36.0%) | 8 (47.1%) |  |
| University education | 144 (55.2%) | 7 (41.2%) |  |
| **Sabadell** | **n = 267** | **n = 108** |  |
| *Age* |  |  |  |
| Mean (SD) [Range] | 15.5 (0.7) [13.2–17.5] | 14.8 (1.0) [12.6–16.4] | **t(135.89)=−7.27, *p*<.001** |
| *Family affluence* |  |  |  |
| Mean (SD) [Range] | 7.2 (0.3) [6.2–7.8] | 7.1 (0.4) [6.3–7.8] | t(40.94)=−1.65, *p*=.107 |
| *Sex N (%)* |  |  |  |
| Female | 134 (50.2%) | 53 (49.1%) | *χ*^2^(1)=0; *p*=.935 |
| Male | 133 (49.8%) | 55 (50.9%) |  |
| *Maternal Education N (%)* |  |  |  |
| Primary or no education | 62 (23.2%) | 28 (29.8%) | *χ*^2^(2)=2.46; *p*=.293 |
| Secondary education | 110 (41.2%) | 31 (33.0%) |  |
| University education | 95 (35.6%) | 35 (37.2%) |  |
| **Valencia** | **n = 221** | **n = 79** |  |
| *Age* |  |  |  |
| Mean (SD) [Range] | 15.5 (0.4) [14.7–17.0] | 15.6 (0.5) [14.7–17.2] | t(89.05)=1.04, *p*=.303 |
| *Family affluence* |  |  |  |
| Mean (SD) [Range] | 7.1 (0.3) [6.4–7.8] | 6.9 (0.2) [6.5–7.6] | **t(184.95)=−4.98, *p*<.001** |
| *Sex N (%)* |  |  |  |
| Female | 116 (46.2%) | 34 (43.0%) | *χ*^2^(1)=0.07; *p*=.799 |
| Male | 135 (53.8%) | 45 (57.0%) |  |
| *Maternal Education N (%)* |  |  |  |
| Primary or no education | 50 (22.6%) | 6 (13.3%) | *χ*^2^(2)=5.12; *p*=.077 |
| Secondary education | 88 (39.8%) | 26 (57.8%) |  |
| University education | 83 (37.6%) | 13 (28.9%) |  |

*Note.* The table shows age (in years), family affluence (range, mean and standard deviation; SD), sex (female, male) and maternal education (primary or no education, secondary education, university education) for participants at Time 1 and Time 2 in each region. The last column presents t-test and χ2 test statistics for sample differences between included and excluded participants. Significant differences are presented in bold. In general, included participants were older at both time points and had higher maternal education at Time 1 in Sabadell, and had higher family affluence at Time 2 in Valencia. Note that participants with missing data for age, family affluence, sex and maternal have been added to the total count of excluded participants per region, but are not included in reported ranges, means and standard deviations and percentages.

**Table S3**

|  | **Returned** | **Did not return** | **Difference** |
| --- | --- | --- | --- |
| **Gipuzkoa (N = 358; 9–12 years)** | **n = 257** | **n = 101** |  |
| *Age* |  |  |  |
| Mean (SD) [Range] | 10.7 (0.2) [10.2–11.4] | 10.7 (0.3) [10.2–11.7] | **t(158.02)=2.18, *p*=.031** |
| *Family affluence* |  |  |  |
| Mean (SD) [Range] | 7.3 (0.3) [6.6–7.9] | 7.2 (0.3) [6.5–7.7] | **t(173.03)=−2.25, *p*=.026** |
| *Sex* |  |  |  |
| Female N (%) | 147 (57.2%) | 48 (47.5%) | *χ*^2^(1)=2.36, *p*=.125 |
| Male N (%) | 110 (42.8%) | 53 (52.5%) |  |
| *Maternal Education* |  |  |  |
| Primary or no education N (%) | 23 (8.9%) | 10 (9.9%) | *χ*^2^(2)=2.37, *p*=.305 |
| Secondary education N (%) | 94 (36.6%) | 45 (44.6%) |  |
| University education N (%) | 140 (54.5%) | 46 (45.5%) |  |
| **Sabadell (N = 385; 12–17 years)** | **n = 265** | **n = 120** |  |
| *Age* |  |  |  |
| Mean (SD) [Range] | 11.2 (0.5) [9.9–12.4] | 11.1 (0.5) [10.0–12.4] | t(231.11)=−0.52, *p*=.605 |
| *Family affluence mean (SD)* |  |  |  |
| Mean (SD) [Range] | 7.2 (0.3) [6.2–7.8] | 7.0 (0.3) [6.3–7.8] | **t(234.15)=−4.01, *p<*.001** |
| *Sex* |  |  |  |
| Female | 133 (50.2%) | 54 (45.0%) | *χ*^2^(1)=0.69, *p*=.405 |
| Male | 132 (49.8%) | 66 (55.0%) |  |
| *Maternal Education N (%)* |  |  |  |
| Primary or no education | 61 (23.0%) | 41 (34.2%) | ***χ*^2^(2)=7.73, *p*=.021** |
| Secondary education | 110 (41.5%) | 51 (42.5%) |  |
| University education | 94 (35.5%) | 28 (23.3%) |  |

**Demographics of participants who returned and did not return at Time 2 (attrition)**

*Note.* The table shows age (in years), family affluence (range, mean and standard deviation; SD), sex (female, male) and maternal education (primary or no education, secondary education, university education) for participants at Time 1 in Gipuzkoa and Sabadell. The last column presents t-test and χ2 test statistics for sample differences between those who returned and those who did not return at Time 2. Significant differences are presented in bold. In general, participants who returned had higher family affluence and were younger in Gipuzkoa, and higher family affluence and maternal education in Sabadell. Note that participants from Valencia did not have data for Time 1.

**SM2. Residential greenness: further details**

Residential greenness was calculated using the Normalized Difference Vegetation Index (NDVI). NDVI was derived from Landsat surface reflectance dataset (Landsat Level- 2 Surface Reflectance Science Product courtesy of the U.S. Geological Survey, (Masek et al. 2006), (Vermote et al. 2016)) from Landsat 4–5 Thematic Mapper (TM) (Landsat 4: 1982 - 1993 and Landsat 5: 1984 - 2011), Landsat 7 Enhanced Thematic Mapper Plus (ETM+) (Landsat 7: 1999 - 2022), and Landsat 8 Operational Land Imager (OLI)/Thermal Infrared Sensor (TIRS) (Landsat 8: 2013 - Ongoing) with 30m x 30m resolution. The highest quality datasets were selected: Level 2, Collection 2, Tier 1. Google Earth Engine (Gorelick et al. 2017) was used to make the image selection (according to cohort years and extent), pixels selection (no negative values, clouds, cloud shadows, water, snow) and to obtain the raw data.

**SM3. Direct Acyclic Graph (DAG)**

Each model described in the main analyses section included a minimum covariate adjustment set derived from a robust causal inference methodology^1,2^, which has previously been applied to the INMA dataset^3^. First, a DAG was designed including measures of interest and relevant covariates, as determined by the theoretical framework. The DAG was then validated in two steps: given the categorical and continuous nature of our measures, we first computed the polychoric correlation matrix of the dataset using the lavCor() function of the *lavaan* package (version 0.6-18)^4,5^. Participants with missing data were excluded from analyses as this function requires datasets with complete cases. We used the localTests() function of the *dagitty* package (version 0.3-4)^1^ to inspect testable implications in this matrix (i.e. pairwise marginal and conditional independencies implied by the DAG)^2^. Testable implications were considered unmet when the correlations were *r*>.20 and *p*<.05. The final correlation matrix including statistics for testable implications is available in Table S4. Unmet implications were taken as an indication of missing relationships and were therefore included in the final validated DAG^6^ (see Figure S2 and Figure S3 for first and validated DAGs, respectively). Table S5 shows minimum covariate adjustment sets for each model as determined by the validated DAG.

**Table S4**

**Testable implications for the validated DAG**

| **Testable implications** | ***r*** | ***p-*value** | **Lower CI**  **(2.5%)** | **Upper CI**  **(97.5%)** |
| --- | --- | --- | --- | --- |
| age ⊥ green \| NDVI, popdens | .08 | .004 | .02 | .13 |
| age ⊥ IMD \| NDVI, popdens | .07 | .005 | .02 | .12 |
| age ⊥ MEDU \| NDVI, popdens | 0 | .924 | -.05 | .05 |
| age ⊥ family affluence \| NDVI, popdens | -.01 | .703 | -.06 | .04 |
| age ⊥ sex | -.01 | .843 | -.06 | .05 |
| green ⊥ sex | -.01 | .698 | -.06 | .04 |
| IMD ⊥ sex | .03 | .244 | -.02 | .08 |
| MEDU ⊥ PS \| age, FS, green, IMD, NDVI, popdens, sex | -.01 | .903 | -.05 | .05 |
| MEDU ⊥ sex | -.02 | .552 | -.07 | .04 |
| NDVI ⊥ sex | 0 | .937 | -.05 | .05 |
| popdens ⊥ sex | -.01 | .567 | -.07 | .04 |
| peer support ⊥ family affluence \| age, FS, green, IMD, NDVI, popdens, sex | .01 | .759 | -.04 | .06 |
| family affluence ⊥ sex | -.04 | .143 | -.09 | .01 |

*Note.* This table shows the testable implications polychoric correlation matrix for the validated DAG. Green = green space availability, NDVI = residential greenness, popdens = population density, IMD = neighbourhood deprivation, MEDU = maternal education, PS = peer support, FS = family support & autonomy.


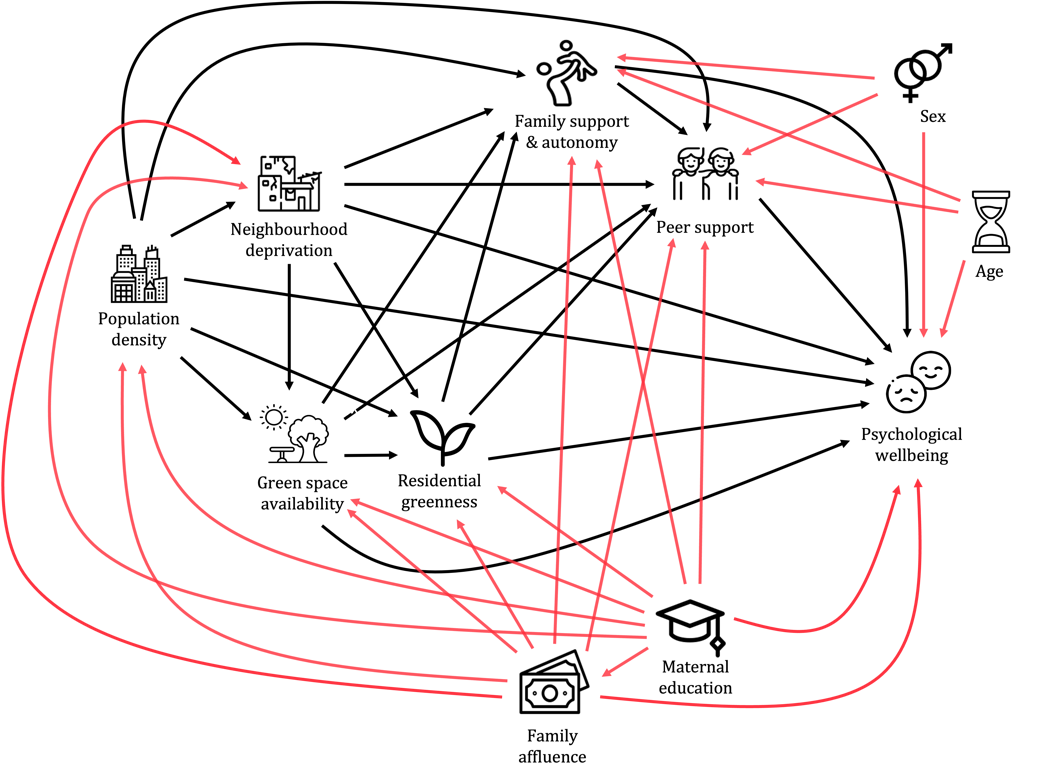


**Figure S2.** **First DAG explaining the relationship between neighbourhood risk and protective factors, perceived** **social support and psychological wellbeing.** Based on previous literature, the model includes the relationships between the different neighbourhood stressors and resources (population density, neighbourhood deprivation, green space availability and residential greenness), the relationship between the neighbourhood stressors and resources and psychological wellbeing, as well as the indirect relationships through family support & autonomy and peer support (black arrows). In addition, the model includes the relationships between confounders and the variables of interest, including maternal education and family affluence, age and sex of the participant (red arrows).


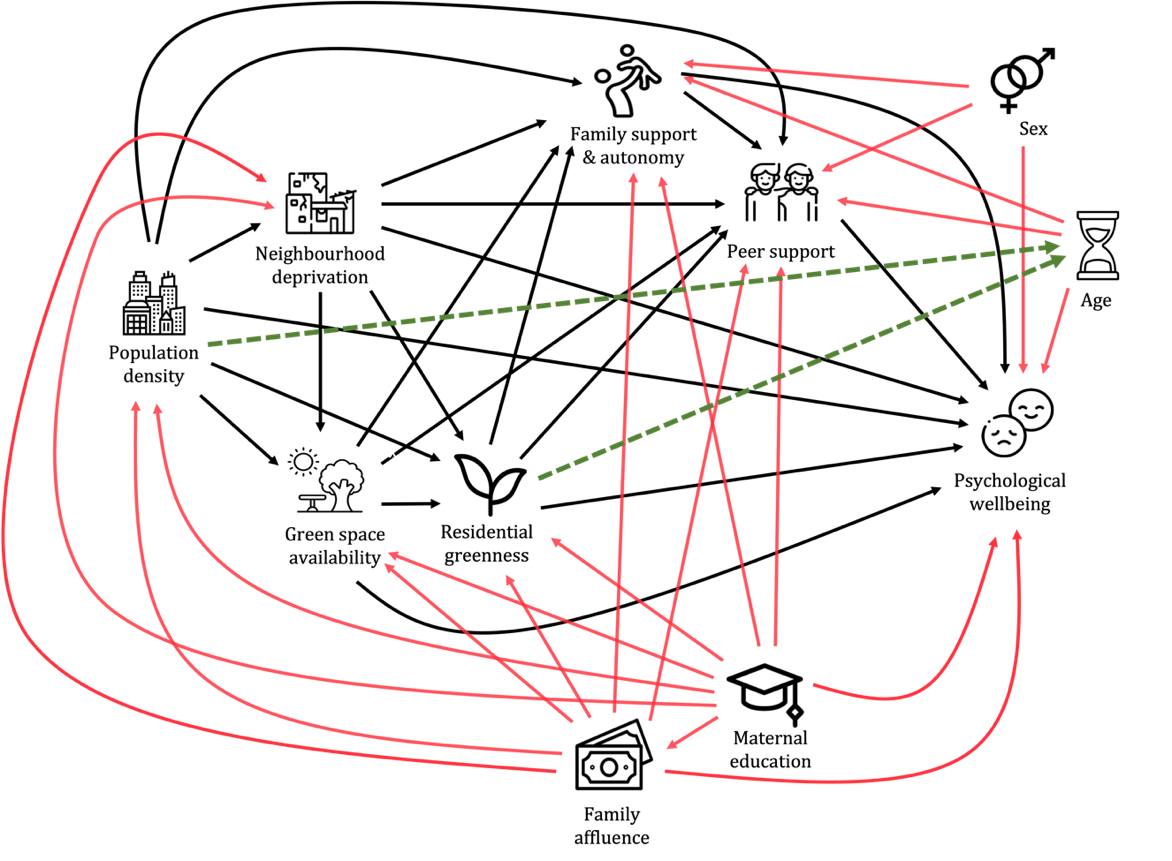


**Figure S3. Validated DAG explaining the relationship between neighbourhood stressors and resources, perceived** **social support and psychological wellbeing.** The validation of the DAG identified the co-dependency between residential greenness and age (correlation *r*=−.30, *p*<.001), and population density and age (correlation *r*=−.23, *p*<.001), and were therefore included in the causal model (green and dotted arrows).

**Table S5**

**Minimum adjustment sets for each model**

| **Predictor** | **Outcome** | **Covariate adjustment set** |
| --- | --- | --- |
| Age | Wellbeing | NDVI, popdens |
|  | Family support & autonomy | NDVI, popdens |
|  | Peer support | NDVI, popdens |
| Family affluence | Wellbeing | MEDU |
|  | Family support & autonomy | MEDU |
|  | Peer support | MEDU |
| Sex | Wellbeing | None |
|  | Family support & autonomu | None |
|  | Peer suppport | None |
| Family support & autonomy | Wellbeing | age, green, IMD, MEDU, NDVI, popdens, family affluence, sex |
| Peer support | Wellbeing | age, FS, green, IMD, NDVI, popdens, sex |
| Residential greenness | Wellbeing | green, IMD, MEDU, popdens, family affluence |
|  | Family support & autonomy | green, IMD, MEDU, popdens, family affluence |
|  | Peer support | green, IMD, MEDI, popdens, family affluence |
| Population density | Wellbeing | MEDU, family affluence |
|  | Family support & autonomy | MEDU, family affluence |
|  | Peer support | MEDU, family affluence |
| Neighbourhood deprivation | Wellbeing | MEDU, popdens, family affluence |
|  | Family support & autonomy | MEDU, popdens, family affluence |
|  | Peer support | MEDU, popdens, family affluence |
| Green space availability | Wellbeing | IMD, MEDU, popdens, family affluence |
|  | Family support & autonomy | IMD, MEDU, popdens, family affluence |
|  | Peer support | IMD, MEDU, popdens, family affluence |

*Note.* This table shows the minimum covariate adjustment set for each model in the analyses, reported in order of appearance in the Results section of the manuscript. Green = green space availability, NDVI = residential greenness, popdens = population density, IMD = neighbourhood deprivation, MEDU = maternal education, FS = family support & autonomy.

**SM5. Results for the relationship between demographics, psychological wellbeing and perceived social support**

The results of linear mixed effects models investigating the relationships between demographic characteristics, psychological wellbeing and perceived social support are presented in Table S6. Summary statistics and post-hoc comparisons of significant main effects showed that psychological wellbeing was lower in older participants (*β*_standardised_=−0.49, 95%CI [−0.54 −0.44], *p*<.001; Figure S4, left panel) and in females compared to males (contrast_Female−Male_=−0.23, SE=0.05, *p*<.001; Figure S4, right panel). In addition, psychological wellbeing was associated with higher family support & autonomy (*β*_standardised_=0.48, 95%CI [0.44 0.52], *p<*.001) and higher peer support (*β*_standardised_=0.28, 95% CI [0.24 0.32], *p*<.001). The relationship between peer support and psychological wellbeing was stronger in females than males (contrast_Female−Male_=0.10, 95% CI [0.02 0.17], *p*=.009) and increased with family affluence (*β*_standardised_=0.06, 95%CI [0.03 0.10], *p<*.001). Finally, older age was related to lower family support & autonomy (*β*_standardised_=−0.08, 95% CI [−0.13 −0.03], *p*=.006) and lower peer support (*β*_standardised_=−0.17, 95% CI [−0.23 −0.12], *p*<.001), and family support & autonomy was weakly lower in females compared to males (contrast_Female−Male_=−0.11, SE=0.05, *p*=.048), however these effects were not robust to sensitivity analyses adjusting for period-specific effects (see Table S7).

**Table S6**

**Results of the linear mixed effects models: psychological wellbeing, social support and demographic characteristics**

| **Outcome measure** | **Predictor measure** | **Effect modification** | **F statistic (DF)** | ***p*-value** |
| --- | --- | --- | --- | --- |
| Psychological wellbeing | Age |  | ***F*(1,561.19)=352.78** | **<.001** |
|  | Sex |  | ***F*(1,876.73)=20.00** | **<.001** |
|  | Family affluence |  | *F*(1,905.35)=0.06 | .806 |
|  | Family support & autonomy |  | ***F*(1,1473.89)=567.35** | **<.001** |
|  |  | Age | *F*(1,1423.51)=1.28 | .257 |
|  |  | Sex | *F*(1,1465.51)=0.85 | .356 |
|  |  | Family affluence | *F*(1,1473.65)=0.06 | .799 |
|  | Peer support |  | ***F*(1,1481.79)=187.19** | **<.001** |
|  |  | Age | *F*(1,1457.90)=0.25 | .616 |
|  |  | Sex | ***F*(1,1478.76)=6.80** | **.009** |
|  |  | Family affluence | ***F*(1,1476.82)=12.27** | **<.001** |
| Family support & autonomy | Age |  | ***F*(1,269.50)=7.61** | **.006** |
|  | Sex |  | ***F*(1,908.43)=3.92** | **.048** |
|  | Family affluence |  | *F*(1,933.55)=0.35 | .556 |
| Peer support | Age |  | ***F*(1,185.24)=33.90** | **<.001** |
|  | Sex |  | *F*(1,892.93)=0.11 | .740 |
|  | Family affluence |  | *F*(1,920.61)=0.08 | .783 |

*Note.* Summary of the linear mixed effects models investigating the relationship between psychological wellbeing (first column) and perceived family support (first column as outcome, second columns as predictors), and demographic characteristics (second column as predictors, third column as modifiers). Significant *F*-tests (fourth column) and associated p­-values (last column) are presented in bold.

~~
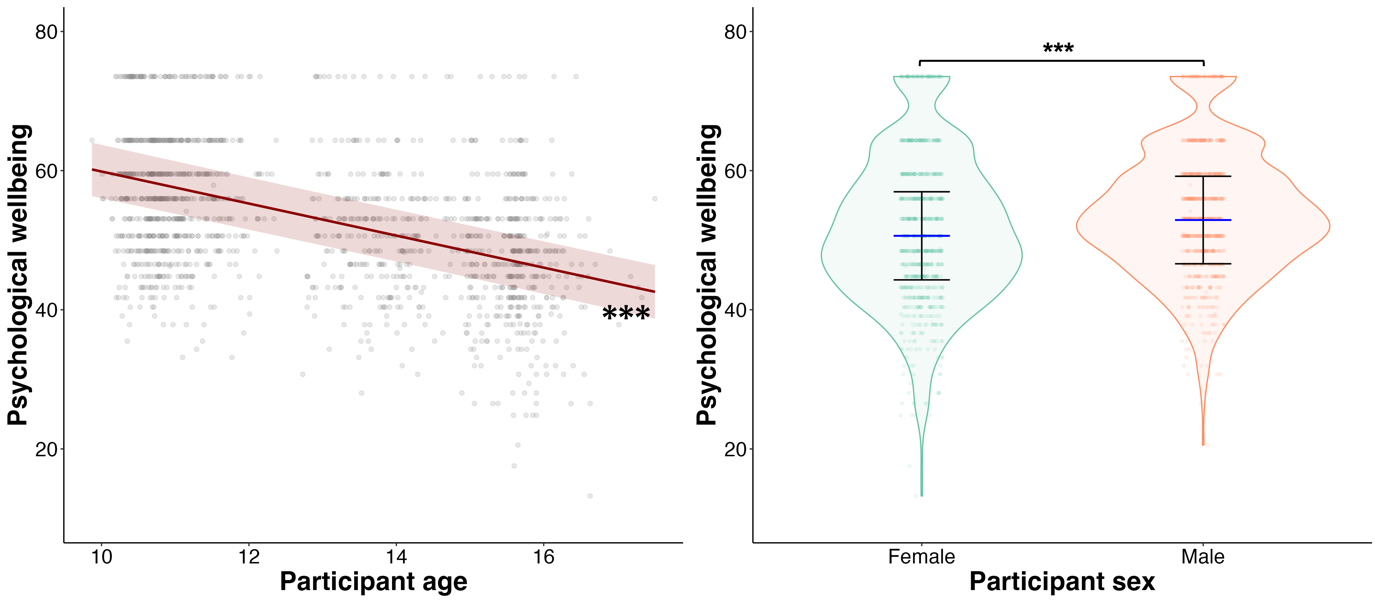
~~

**Figure S4. Age and sex-related differences in psychological wellbeing.** These plot shows the results of the linear mixed effects model for the effect of age (left panel) and sex (right panel) on psychological wellbeing. Dots are individual psychological wellbeing data points per participant age or sex. The line of best fit and shaded area shows the main effect of age and 95% confidence interval (in red, left panel), and line and whiskers show mean psychological wellbeing and 95% confidence interval for females (green, right panel) and males (orange, right panel), as estimated by the linear mixed effects models. The asterisk indicates a significant main effect of age and a significant contrast between females and males: ****p*<.001.

**SM6. Sensitivity analyses of primary analyses excluding influential observations, adjusting for period-specific effects and including sample weights for selective attrition**

Model diagnostics of the linear mixed effects models suggested that data was normally distributed, but that homogeneity of variance and linearity assumptions could be violated towards the extremes of the scales, potentially due to the presence of influential observations. Therefore, in a first set of sensitivity analyses, we re-ran all models excluding potential influential observations (SA1). In addition, to adjust for period-specific influences in the relationships, we re-ran a second set of sensitivity analyses additionally including *timepoint* (0,1) as a fixed effect to all models (SA2). Finally, to adjust for selective attrition (i.e. differences between participants who were included and excluded for missing data at random and attrition), a third set of sensitivity analyses adjust for participant-level weights. These weights were obtained by deriving the inverse probability of inclusion/exclusion predicted by age, family affluence, maternal education, time and region^28^. Table S7 shows model statistics for the significant main effects and interactions reported in the manuscript and after adjusting for SA1, SA2 and SA3. All main effects and interactions remained significant after adjusting for SA1, SA2 SA3, except for the main effect of age and the main effect of sex on family support & autonomy, as well as the main effect of age on peer support.

**Table S7**

**Results of the sensitivity analyses of the linear mixed effects models**

| **Main effects and interactions** | **Main model** | **SA1: Influential observations** | **SA2: Timepoint** | **SA: Weights** |  |
| --- | --- | --- | --- | --- | --- |
| ***Main effects of age on social support and psychological wellbeing*** | | | | | |
| Wellbeing ~ age | *F*(1,561.19)=352.78; *p*<.001 | *F*(1,568.67)=393.89; *p*<.001 | *F*(1,791.12)=46.04; *p*<.001 | *F*(1,589.64)=354.07; *p*<.001 |  |
| Family support & autonomy ~ age | *F(*1,269.50)=7.61; *p*=.006 | *F(*1,292.22)=10.08; *p*=.002 | ***F(*1,182.69)=1.58; *p*=.210** | *F(*1,281.72)=7.87; *p*=.005 |  |
| Peer support ~ age | *F(*1,185.24)=33.90; *p*<.001 | *F(*1,199.33)=34.81; *p*<.001 | ***F(*1,122.26)=3.00; *p*=.086** | *F(*1,205.46)=34.91; *p*<.001 |  |
| ***Main effects of sex on social support and psychological wellbeing*** | | | | | |
| Wellbeing ~ sex | *F*(1,876.73)=20.00; *p*<.001 | *F*(1,866.92)=19.78; *p*<.001 | *F*(1,908.29)=17.61; *p*<.001 | *F*(1,971.93)=21.22; *p*<.001 |  |
| Family support & autonomy ~ sex | *F*(1,908.43)=3.92; *p*=.048 | *F*(1,842.57)=4.47; *p*=.035 | ***F*(1,909.73)=3.73; *p*=.054** | *F*(1,986.25)=4.37; *p*=.037 |  |
| ***Main effects and interactions of social support on psychological wellbeing*** | | | | | |
| Wellbeing ~ family support & autonomy | *F*(1,1473.89)=567.35; *p*<.001 | *F(*1,1465.86)=601.31; *p*<.001 | *F(*1,1472.78)=567.44; *p*<.001 | *F(*1,1620.45)=569.64; *p*<.001 |  |
| Wellbeing ~ peer support | *F*(1,1481.79)=187.19; *p*<.001 | *F*(1,1475.72)=191.32; *p*<.001 | *F*(1,1480.59)=186.91; *p*<.001 | *F*(1,1655.84)=187.54; *p*<.001 |  |
| Wellbeing ~ peer support x sex | *F*(1,1478.76)=6.80; *p*=.009 | *F*(1,1470.53)=8.44; *p*=.004 | *F*(1,1477.73)=6.73; *p*=.010 | *F*(1,1654.10)=6.86; *p*<.001 |  |
| Well-being ~ peer support x family affluence | *F*(1,1476.82)=12.27; *p*<.001 | *F*(1,1470.35)=10.88; *p*=.001 | *F*(1,1475.78)=12.12; *p*<.001 | *F*(1,1686.35)=12.34; *p*<.001 |  |
| ***Main effects and interactions of neighbourhood characteristics on psychological wellbeing*** | | | | | |
| Wellbeing ~ population density x age | *F*(1,759.01)=9.59; *p*=.002 | *F*(1,848.09)=9.80; *p*=.002 | *F*(1,434.76)=8.31; *p*=.004 | *F*(1,816.64)=9.49; *p*=.002 |  |
| Wellbeing ~ residential greenness x age | *F*(1,829.03)=8.95; *p*=.003 | *F*(1,808.18)=10.12; *p*=.002 | *F*(1,1075.28)=7.59; *p*=.006 | *F*(1,894.55)=8.83; *p*=.003 |  |
| ***Main effects and interactions of neighbourhood characteristics on social support*** | | | | | |
| Family support ~ residential greenness | *F*(1,7.96)=5.73; *p*=.044 | *F*(1,7.68)=5.75; *p*=.045 | *F*(1,11.95)=4.96; *p*=.046 | *F*(1,8.07)=5.90; *p*=.041 |  |
| Family support ~ residential greenness x age | *F*(1,760.70)=7.66; *p*=.006 | *F*(1,722.29)= 8.95; *p*=.003 | *F*(1,836.88)= 9.12; *p*=.003 | *F*(1,820.96)= 7.65; *p*=.006 |  |
| Peer support ~ residential greenness x age | *F*(1,780.55)=3.80; *p*=.052 | *F*(1,782.63)=3.34; *p*=.068 | *F*(1,299.09)=4.54; *p*=.034 | *F*(1,847.50)=3.96; *p*=.047 |  |

*Note.* This table shows the main effects and interactions (first column) from the results of the linear mixed effects models reported in the main manuscript (second column), as well as the main effect and interactions after adjusting for influential observations (SA1; third column), after including for *timepoint* as a fixed effect to adjust for period-specific effects (SA2; fourth column), and after included participant-level weights to adjust for selective attrition (SA3; fifth column). Main effects and interactions what were no longer significant after adjusting for the sensitivity analyses are presented in bold.
